# A Systematic Investigation on the Effect of Carbon Nanotubes and Carbon Black on the Mechanical and Flame Retardancy Properties of Polyolefin Blends

**DOI:** 10.3390/polym16030417

**Published:** 2024-02-01

**Authors:** Eid M. Alosime, Ahmed A. Basfar

**Affiliations:** 1 King Abdulaziz City for Science and Technology, P.O. Box 6086, Riyadh 11442, Saudi Arabia; 2 M.Sc. in Nuclear Engineering Program, College of Engineering, King Saud University, Riyadh P.O. Box 145111, Saudi Arabia; abasfar@ksu.edu.sa; 3 Mechanical Engineering Department, College of Engineering, King Saud University, P.O. Box 800, Riyadh 11421, Saudi Arabia

**Keywords:** EVA/LLDPE blend, carbon nanotubes, carbon black, flame retardant, wire and cable insulation, mechanical properties

## Abstract

Due to high filler loading, clean, commercial, thermoplastic, flame-retardant materials are mechanically unstable when insulating wires and cables. In this study, composite formulations of linear low-density polyethylene (LLDPE)/ethylene–vinyl acetate (EVA) containing a flame retardant, such as magnesium hydroxide (MH; formula: Mg(OH)_2_) and huntite hydromagnesite (HH; formula: Mg_3_Ca(CO_3_)_4_, Mg_5_(CO_3_)_4_(OH)_2_·3H_2_O), were prepared. The influence of carbon nanotubes (CNTs) and carbon black (CB) on the mechanical properties and flame retardancy of LLDPE/EVA was studied. Three types of CNTs were examined for their compatibility with other materials in clean thermoplastic flame-retardant compositions. The CNTs had the following diameters: 10–15 nm, 40–60 nm, and 60–80 nm. Optimum mechanical flame retardancy and electrical properties were achieved by adding CNTs with an outer diameter of 40–60 nm and a length of fewer than 20 nm. Large-sized CNTs result in poor mechanical characteristics, while smaller-sized CNTs improve the mechanical properties of the composites. CB enhances flame retardancy but deteriorates mechanical properties, particularly elongation at break, in clean, black, thermoplastic, flame-retardant compositions. Obtaining satisfactory compositions that meet both properties, especially formulations passing the V-0 of the UL 94 test with a minimum tensile strength of 9.5 MPa and an elongation at break of 125%, is challenging. When LLDPE was partially substituted with EVA, the limiting oxygen index (LOI) increased. The amount of filler in the formulations determined how it affected flammability. This study also included a reliable method for producing clean, black, thermoplastic, flame-retardant insulating material for wire and cable without sacrificing mechanical properties.

## 1. Introduction

Electrical wiring-related fires cause significant human and material losses worldwide because of high-temperature flames, poisonous smoke, and gas from combustible insulation materials. With the increasing number of people in densely populated buildings, poor insulation in wires and cables increases the risk of death and property damage. Polyethylene (PE) and polyvinyl chloride (PVC) compounds are excellent insulations, but their lack of flame retardancy and excessive production of poisonous gases pose significant problems. PE is flammable but produces less toxic gases during burning, while PVC generates high levels of poisonous gases. Researchers are exploring flame-retardant materials like halogen-free compounds, clean flame-retardant materials, and nontoxic materials [1,2,3,4,5]. Pure materials contain halogen and toxicity-free chemicals but have poor mechanical properties. Commercial clean materials with high flame retardancy often have unstable mechanical properties due to their high filler loadings. High levels of flame retardants have the potential to deteriorate mechanical properties significantly. Tensile strength, elongation at break, thermal resistance, and flame retardancy requirements should all be met by the materials used for insulation and jackets.

Because of their excellent flame-retardant qualities and load capabilities, ethylene–vinyl acetate (EVA), ethylene–vinyl acetate/LDPE (low-density PE), ethylene alpha-olefin, and ethylene ethyl acrylate are commonly utilized as matrix polymers. The high decomposition temperature and smoke suppression capabilities of inorganic compounds, such as aluminum hydroxide, magnesium hydroxide (MH), and huntite hydromagnesite (HH), render them flame retardants [6,7,8,9]. Encapsulated organic flame retardants enhance interfacial adhesion and dispersion, but hydrotalcite composites release more gas during a fire [10]. The fire properties of organo-modified montmorillonite are improved when it is partially substituted for conventional flame retardants [11]. Nonetheless, a minimum tensile strength, elongation at break standards, and superior mechanical and high flame-retardant properties must be met. A pure flame-retardant material with superior mechanical properties is required. Electrically conductive polymers like polypyrrole and polyaniline are expensive and difficult to process [12]. Conductive fillers are often introduced into the polymer matrix to improve their electrical conductivity, but the final properties of these composites depend on their filler content. Maintaining balanced mechanical and processing properties while improving the electrical and thermal conductivity of the polymer matrix is crucial for these properties. The percolation threshold is the critical value at which the filler content’s behavior changes from insulating to conducting. Improving mechanical properties depends on the share of particles, their proper distribution, and the morphology of the polymer matrix [13].

Due to their unique thermal, mechanical, and electrical properties, including tunable conductivity, composites containing conductive fillers like carbon black (CB), carbon nanotubes (CNTs), or graphene dispersed in insulating polymers can be used in a wide range of industrial applications. Conductive fillers are typically added in enormous concentrations to achieve significant electrical conductivity in many applications, resulting in diminished mechanical properties, increased melt viscosity, and higher expenses. CNTs are excellent candidates for replacing or complementing conventional fillers in the production of multifunctional polymer composites, owing to their unique combination of mechanical, electrical, and thermal properties [14,15,16]. CNTs can enhance the electrical conductivity of insulating polymers at relatively low concentrations compared to CB. Compared to more traditional fillers like CB, CNTs have a higher aspect ratio (L/D), a significant factor in their emergence as effective fillers [17,18,19]. Li et al. [20] investigated the influence of CB on high-density PE (HDPE) composites with propylene–ethylene random copolymer elastomers. The results showed that fillers improved the viscoelasticity, conductivity, and surface resistance. CB with a content of over 5 wt% showed a nucleation effect, improved HDPE crystallinity and stiffness, and decreased ductility. Ahmed et al. [21] studied the effect of electron beam irradiation and CB filler loading on the properties of LLDPE related to changes in mechanical properties and phase morphology, and found that irradiated CB-filled LLDPE had higher mechanical properties than unirradiated LLDPE. The morphology exhibited a smooth structure that gradually shifted from being ductile to less flexible and rigid as the CB filler loading and radiation dose increased. Nevertheless, the elongation at break decreased with increasing CB filler loading for the irradiated LLDPE. The fire-retardant characteristics of LLDPE/CNT were studied by Bocchini et al. [22], who showed that the thermal stabilization of LLDPE/CNT nanocomposites can be achieved with a low concentration of CNTs (0.5%) due to the surface effect formed during the first step of LLDPE volatilization. Liu et al. [23] studied the impact of halogen-free flame-retardant blends of LLDPE/ethylene–acrylic acid copolymer (EAA)/MH composites on their properties. They found that EAA increases the limiting oxygen index (LOI) and reduces heat release and smoke production rates. It also enhances thermal oxidative stability by creating a stable barrier that prevents heat and mass transfer during fires. Additionally, EAA increases the elongation at break and impact strength of composites. Xu et al. [24] also assessed CNTs wrapped in an intumescent flame retardant. The diameter of the CNTs was controlled to 20–90 nm, and they were better dispersed in polypropylene (PP) due to in situ compatibilization. This approach enhances the flame retardancy and mechanical properties of polymeric materials.

CB is well known to increase flame retardancy but deteriorate mechanical properties, especially the elongation at break in black-colored, thermoplastic, clean, flame-retardant compositions. This seminal work has inspired researchers to explore a substitute material for CB that does not deteriorate mechanical properties and has improved flame retardancy in thermoplastic, clean, flame-retardant compositions. This study intends to find the best compromise between good mechanical performance, easy processability, and adequate flame-retardant characteristics in composite materials. The main limitation of halogen-free flame retardants (HFFRs) is their excessive content within the polymer matrix. An EVA copolymer was chosen due to its high amorphous content, allowing for extensive filler content [25]. The formulations were tested using a blend of EVA with 20% and 80% LLDPE and the type of conductive filler. Evaflex 360 was chosen due to its thermal degradation process, resulting in a protective crust on the cable surface, making EVA a popular choice for cable production [26]. EVA’s heat-at-combustion value is also lower than that of apolar polyolefins like PE [25]. Two grades of MH and HH were combined with other secondary flame retardants to identify the finest trade-off between composite flame-retardant performance, mechanical properties, and manufacturability. The following are the primary criteria for selecting the new material for this work:

- The material must be a novel substance that may be utilized in thermoplastic, clean, flame-retardant compositions instead of CB to increase flame retardancy;
- The material’s mechanical properties, particularly elongation at break, may be improved;
- The material must be compatible with the other components of the flame-retardant mixture.

## 2. Experimental Section

### 2.1. Materials

The basis polymers were linear low-density polyethylene (LLDPE 118W, melt flow index: 1.0 g/10 min, SABIC, Riyadh, Saudi Arabia) and ethylene–vinyl acetate (Evaflex 360, vinyl acetate content: 25%, melt mass-flow rate: 190 °C/2.16 kg: 2.0 g/10 min, DuPont-Mitsui Polychemicals Co., Tokyo, Japan). Carbon black (Corax N550, semi-active carbon black with high structure, ash content: 0.5% Degussa, Frankfurt, Germany), CM-95 carbon nanotubes (diameter 10–15 nm and length 10–20 µm, Hanwha Nanotech, Seoul, Republic of Korea), CNT50 carbon nanotubes (inner diameter: 10–30 nm, outer diameter: 40–60 nm, length distribution: <20 μm, NanoKarbon Co., Ltd., Seoul, Republic of Korea), and CNT75 carbon nanotubes (inner diameter: 30–50 nm, outer diameter: 60–80 nm, length distribution: <20 μm, NanoKarbon, Korea Nano Ind. Co.) were used as fillers (details of SEM micrographs can be found in Supplementary Materials, Figure S1). Magnifin A-H10A magnesium hydroxide (Albemarle, Paris, France), KISUMA 5B magnesium hydroxide (Kyowa Chemical, Sakaide, Japan), and huntite hydromagnesite (Ultracarb LH15X, Minelco, Cincinnati, OH, USA) were used as the primary flame retardants. A red phosphorus (RP) masterbatch (Exolit RP 692, phosphorus content: approx. 50% (*w*/*w*), Clariant, Cergy, France), zinc borate (ZB) (Firebrake ZB, melting point: phase change at 650 °C, Borax, Boron, CA, USA), and boric acid (BC) (melting point: 170 °C, boiling point: 300 °C, Rose Mill Chemicals & Lubricant, USA) were used as secondary (intumescent) flame retardants. Pentaerythritol tetrakis(3(3,5-di tert-buty-4-hydroxyphenyl) propionate (Irganox1010, CIBA Specialty Chemicals, Basel, Switzerland) was used as an antioxidant. No materials underwent additional purification; all were of commercial grade.

### 2.2. Methods

EVA and LLDPE were melted and mixed at 150 °C for four minutes in an internal mixer 350S (Brabender Co., Duisburg, Germany). The remaining fillers and flame retardants were blended with the previously melted polymers for 10 min at 150 °C. The pre-blended compounds were pelletized in a two-roll mill/guider cutter/pelletizing extruder. The temperature of the two-roll blender was held at around 150 °C throughout this step, and the mixture was processed for 5–10 min. The mixture was transferred to a hydraulic hot press and compressed for 20 min at 165 °C. The test specimens were cut into sheets with dimensions of 110 mm and 185 mm and a thickness of 2 mm. Hot compression was performed for 10 min at 150 °C.

### 2.3. Characterizations

The tensile strength and elongation at break were measured at room temperature (25 °C) using a universal testing machine from Instron, Norwood, MA, USA, at a speed of 500 mm per minute at 25 °C. The samples were thermally aged for 168 h in an oven at 100 °C.

The flammability of the produced formulations was assessed using the UL-94 and LOI flammability tests. A flammability chamber from CEAST Co., Turin, Italy, was used to conduct UL-94 flammability tests in line with ASTM D635 for the horizontal position and ASTM D3801 for the vertical position [27,28]. The LOI test was conducted in line with ISO 4589 (ASTM D2863) using a device from Fire Testing Technology Limited (Incorporating Stanton Redcroft, London, UK) [29]. The LOI is equivalent to the lowest oxygen content (80 × 10 × 1) required for specimen combustion in an oxygen–nitrogen environment.

Volume resistivity was determined at 25 °C on a high-resistance meter model HP4339B from HP, Palo Alto, CA, USA, by ASTM D257 [30].

## 3. Results and Discussion

In the significant specification of thermoplastic, HFFR-insulated cables, such as IEC 60502 or BS 6724, HFFR compounds are used in jacket materials. Most jacket colors of volt power cables are black and contain CB. The prominent role of CB in polymer composites is to absorb UV and increase flame retardancy. CNTs may be utilized instead of CB in thermoplastic, clean, flame-retardant compositions to improve their mechanical performance and flame retardancy. CNTs are ideal for this application. Other CNT-compatible flame-retardant compositions include MH and HH. These components have a fine structure and can be used in flame-retardant formulations.

### 3.1. Effects of CB Contents on Flame-Retardant EVA/LLDPE Composites

The effects of CB on various types of flame-retardant materials were investigated. This study revealed that thermoplastic, clean, flame-retardant compositions, including a main flame-retardant MH (MAGNIFIN A-H10A) grade and various CB contents (Table 1), showed a slight decrease in elongation at break and an increase in flame retardancy by 25.80% with an increase in the CB content. A similar tendency was observed when the primary flame retardant was changed from MH (MAGNIFIN A-H10A) to HH (Ultracarb LH15X), as shown in Table 2. It was observed that the elongation at break decreased slightly, although the tensile strength and flame retardancy increased with the increased CB content. The presence of the filler results in a less complicated break formation process due to increased stress on the filler surface and, on the other hand, a decrease in chain mobility because of the polymer–filler interface, which lowers the combinations’ deformability. The elongation-at-break value is reduced as a result of these effects [31,32]. When MH (KISUMA 5B) grade was present, the tensile strength did not significantly alter when the CB content increased. Conversely, there was a noticeable effect of CB on the elongation at break. As the CB content increased, the elongation at break decreased (Table 3). Generally speaking, a higher filler content can result in worse mechanical properties. Although a CB filler was used in the compounds, their tensile strength stayed nearly constant, and their elongation at break was significantly affected below 8 wt% of CB.

Furthermore, the LOI (%) increased as the CB content increased. Our investigation shows that, in black, thermoplastic, clean, flame-retardant compositions, CB increases flame retardancy and decreases elongation at break. Following the above discussion, the results show that, according to IEC 60502 standards [33], no composites pass the V-0 of the UL-94 test with a minimal tensile strength of 9.5 MPa and a minimal elongation at break of 125%. A higher than 120 phr content of the primary flame retardant plus secondary (intumescent) flame retardants like RP, ZB, and BA must be compounded to pass the UL 94 test’s V-0. Table 4 illustrates that even with increased flame retardancy, extra main and intumescent flame retardants may result in a decline in mechanical characteristics. Despite having high LOI values, formulations of C-17 to C-20 that contained CB exhibited a significant decrease in elongation at break. Many scientists are working to find a CB replacement with better flame retardancy in thermoplastic, clean, flame-retardant compositions without their sacrificing mechanical properties [34,35,36]. For this specific goal, we introduced a novel material in our study that can replace CB in thermoplastic, clean, flame-retardant compositions.

### 3.2. Effects of CNT and CB Contents on Flame-Retardant EVA Composites

The relationships between each flame retardant and CNT/CB were investigated. The correlations between MH (Magnifin A-H10A) grade and CNT/CB were explored as a preliminary test, as shown in Table 5. Because of the low-percolation-threshold requisite for high-aspect-ratio fillers, conducting polymer structures were created at low-CNT filler loading levels [37]. The characteristic values were fewer than 6 phr CNT loadings compared to 2–6 phr CB components. Low particle components are essential for electronic clean-room applications (where particle contamination is a severe issue).

**Figure 1:**
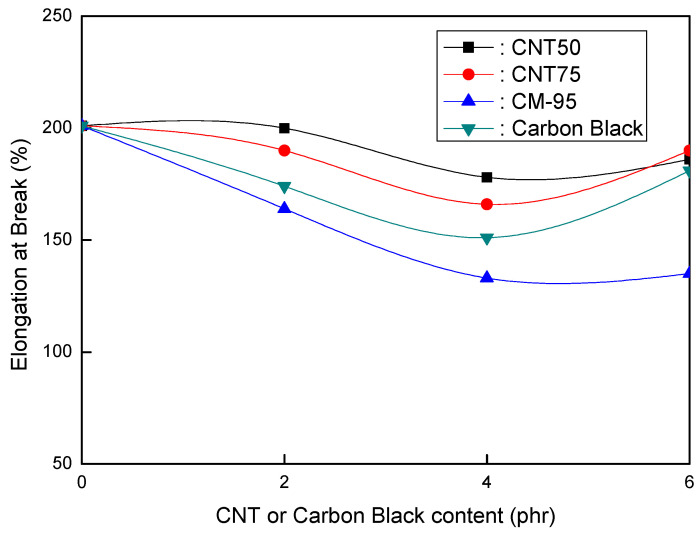
Elongation at break (%) of EVA/120 phr MH (Magnifin A-H10A) formulations as a function of CNT or CB content.

**Figure 2:**
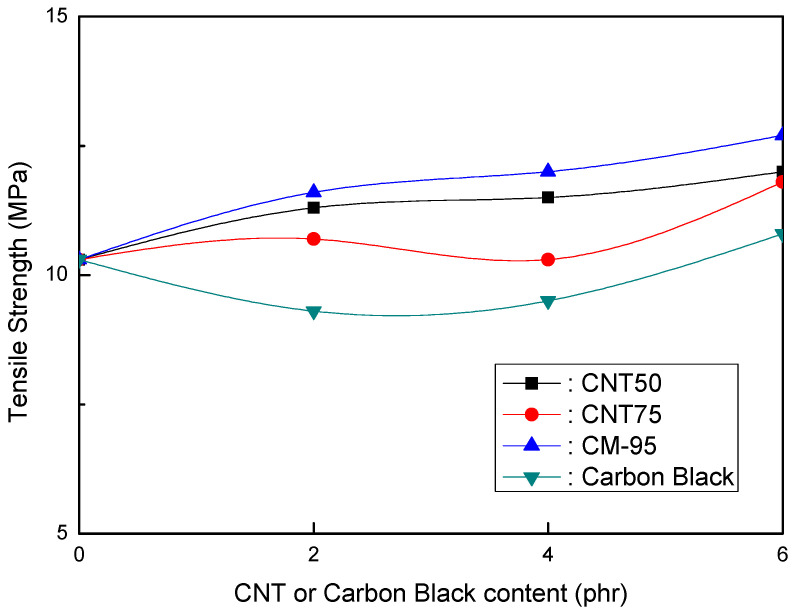
Tensile strength (MPa) of EVA/120 phr MH (Magnifin A-H10A) formulations as a function of CNT or CB content.

**Figure 3:**
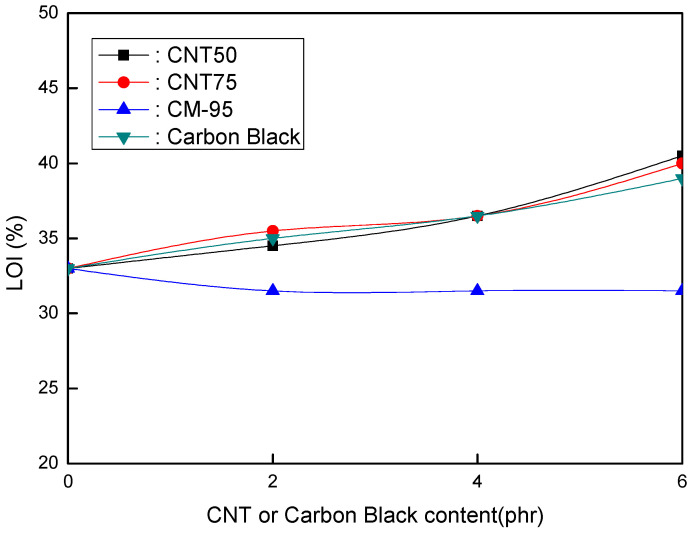
LOI (%) of EVA/120 phr MH (Magnifin A-H10A) formulations as a function of CNT or CB content.

Figure 1, Figure 2 and Figure 3 compare CNT and CB in EVA/120 phr MH (Magnifin A-H10A) formulations. Figure 1 illustrates the relationship between the elongation at break and CNT or CB content for EVA/120 phr MH (Magnifin A-H10A) formulations. Even though the content was small, different trends from other CNTs were seen. The elongation at break reduced with higher CNT contents at ranges of up to 4 phr. When compared to CB, CM-95 exhibited the lowest elongation at break. The size of the particle is thought to affect its mechanical properties. When elongation at break is examined between CNT50/CNT75 formulations and formulations including CB, it is clear that CNT50/CNT75 formulations exhibit greater values. CNT50/CNT75 may be used in HFFR formulations instead of CB to attain more excellent elongation at break values.

The tensile strength of EVA/120 phr MH (Magnifin A-H10A) formulations is plotted against the CNT or CB content in Figure 2. Even with a low content, different trends from other CNTs were seen. The tensile strength increased as the CNT content increased to a range of up to 6 phr. CM-95 exhibited the most substantial tensile strength compared to formulations containing CB. According to one theory, smaller particles may show greater tensile strength. As a result, CB had the lowest tensile strength, while CM-95 had the strongest. Tensile strength comparisons between the CNT50/CNT75 formulations and CB-containing formulations revealed that the CNT50/CNT75 formulations exhibited significantly higher values.

Figure 1 and Figure 2 show that the CNT50/CNT75 formulations have higher values for both mechanical properties (elongation at break and tensile strength) than the CB-containing formulations. CNT50/CNT75 can be used in HFFR formulations instead of CB to provide more excellent mechanical properties (especially elongation at break). The stress intensity on the CB filler surface might cause small faults that lead to the breakdown of filled combinations with the polymer matrix. As a result, little fractures begin to form and grow until they reach the critical crack level. The formation and propagation of cracks in the same material without filler are accidental, and the collapse manifests at a reasonably high distortion. However, the presence of filler simplifies break formation due to increased stress and decreased chain mobility caused by the polymer–filler interface, reducing the combinations’ deformability and elongation-at-break values [38,39].

Figure 3 depicts the LOI (%) of EVA/120 phr MH (Magnifin A-H10A) formulations as a CNT or CB concentration function. The CNT50, CNT75, and CB formulations had identical flame retardancy trends as a content function. However, the CM-95 formulation had very low flame retardancy. Increasing concentrations improved the flame retardancy of the CNT50, CNT75, and CB formulations. Flame retardancy increases even with a 2 phr content of CNT50, CNT75, or CB. It is considered that these materials have a strong flame-retardant power with a combination of MH (Magnifin A-H10A), except CM-95. To slow down the polymer degradation rate, the polymer/CNT network structure layer served as a shield, reflecting incident radiation into the gas phase. Therefore, low loadings are required to achieve meaningful fire retardancy. Unfavorable alterations to the mechanical and physical properties of polymers can be prevented by using appropriate comonomer selection or other modifying groups. Reactive monomers and naturally flame-resistant polymers made of P, Si, N, Bi, and other random elements are examples of current advancements in HFFR polymers [40].

The findings shown in Figure 1, Figure 2 and Figure 3 clarify that the CNT50/CNT75 formulations outperformed the CB-containing formulations with regard to their mechanical properties without sacrificing flame retardancy. Increased mineral loading led to a decrease in the mechanical potential of the components [41]. Kashiwagi et al.’s [42] initial introduction of CNTs into PP was to reduce its flammability behavior. It was determined that the growth of the CNT structural network layer, which might protect the underlying polymer, was the most likely cause of the decrease in the heat release rate.

The relationships between each flame retardant and CNT/CB are continuous. The relationships between MH (KISUMA 5B) grade and CNT/CB are shown in Table 6.

**Figure 4:**
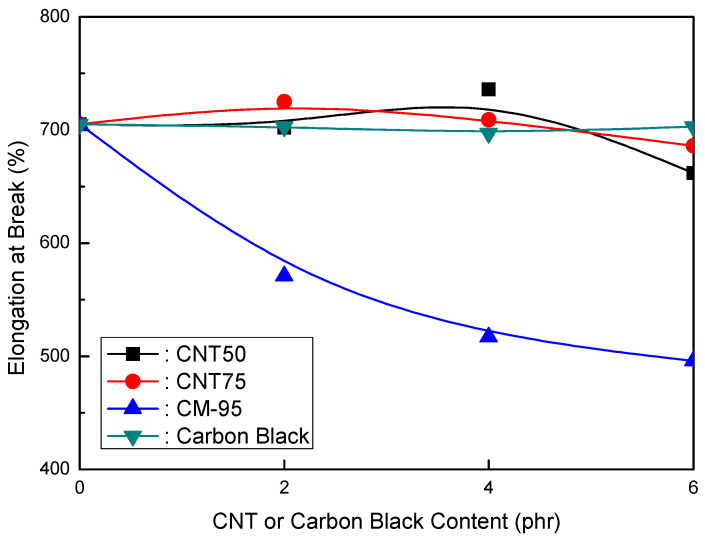
Elongation at break (%) of EVA/120 phr MH (KISUMA 5B) formulations as a function of CNT or CB content.

**Figure 5:**
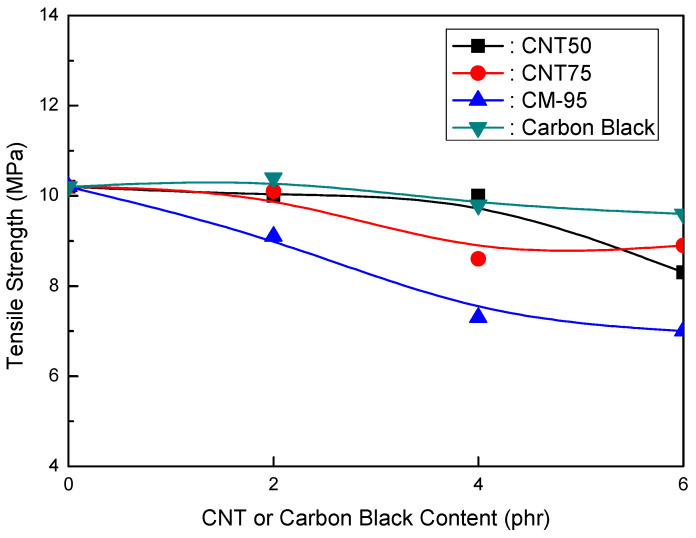
Tensile strength of EVA/120 phr MH (KISUMA 5B) formulations as a function of CNT or CB content.

The elongation at break and tensile strength of EVA/120 phr MH (KISUMA 5B) grade formulations as a CNT or CB content function are shown in Figure 4 and Figure 5. Like MH (Magnifin A-H10A) grade formulations, different trends were found from other CNTs, although the content was relatively low. At a range of up to a 4 phr content, in the CNT50, CNT75, and CB formulations, the elongation at break did not change with an increase in the CNT content. On the contrary, in the CM-95 formulation, the elongation at break essentially decreased with the increase in content. Almost identical results were observed in the tensile strength tests. The effects of the mechanical properties of the MH Magnifin A-H10A formulations were different from those of the MH KISUMA 5B formulations. The results of the CM-95 formulation were different from those of the CNT50, CNT75, and CB formulations. However, MH KISUMA 5B is a synthetic MH product with a higher fatty acid content on the surface. The size distribution of the particles remained unchanged with the application of an organic agent. Nevertheless, it did cause a significant decrease in the specific surface area of 5–7 m^2^/g without causing particle aggregates to form. The brucite surface’s clogged pores likely caused the surface area to decrease, which may have led to lower tensile strength values and increased elongation at break. According to Haveriku et al. [43], fatty acid-based thermoplastic treatments often increase elongation and impact resistance while decreasing modulus, tensile strength, filler dispersion, and melt viscosity.

**Figure 6:**
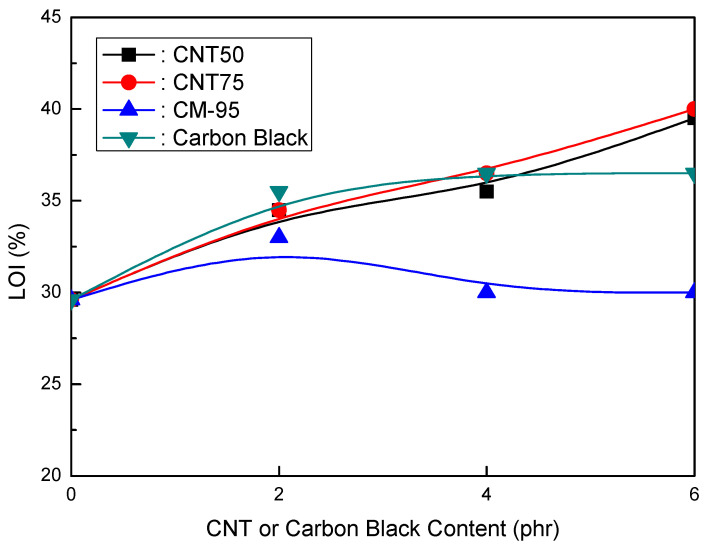
LOI (%) of EVA/120 phr MH (KISUMA 5B) formulations as a function of CNT or CB content.

The LOI (%) of EVA/120 phr MH (KISUMA 5B) formulations as a function of the CNT or CB content is shown in Figure 6. Like the MH (Magnifin A-H10A) grade formulations, the CNT50, CNT75, and CB formulations showed almost the same trends of flame retardancy as a content function, whereas the CM-95 formulation showed very low flame retardancy. Flame retardancy increased with the increase in the CNT50, CNT75, and CB formulations, while flame retardancy did not change with the rise in the CM-95 content. The lowest LOI was found for CM-95, which has a smaller particle size.

The relationships between each flame retardant and CNT/CB are continuous. The relationships between HH (Ultracarb LH15X) and CNT/CB are shown in Table 7.

**Figure 7:**
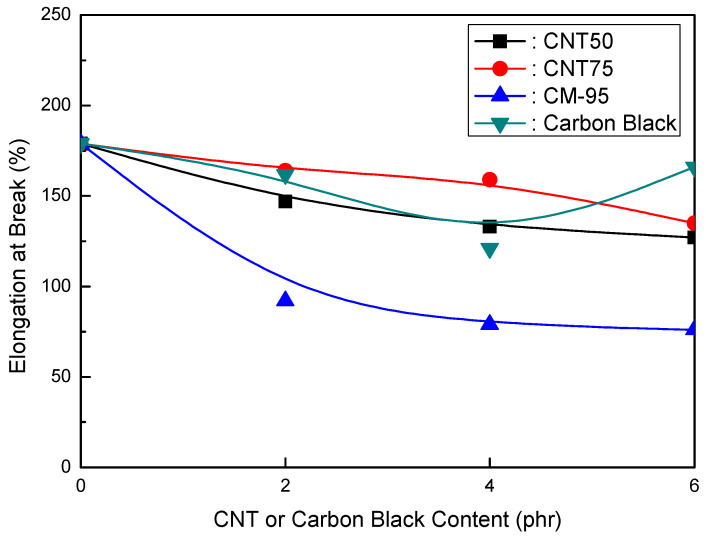
Elongation at break (%) of EVA/120 phr HH (Ultracarb LH15X) formulations as a function of CNT or CB content.

**Figure 8:**
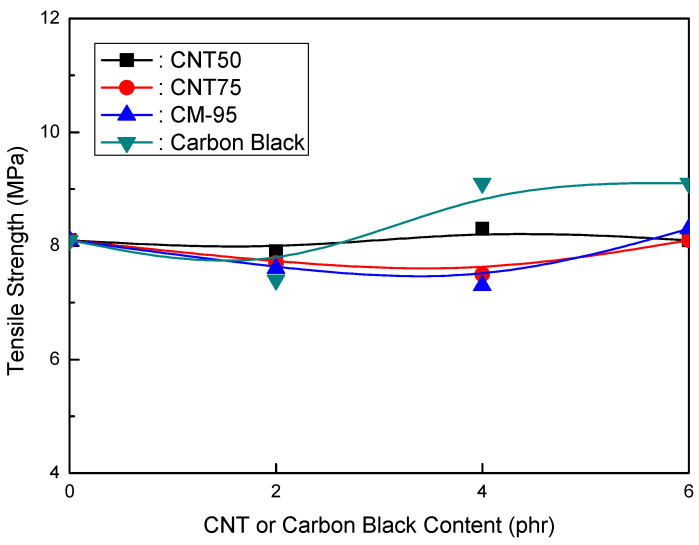
Tensile strength of EVA/120 phr HH (Ultracarb LH15X) formulations as a function of CNT or CB content.

Figure 7 and Figure 8 demonstrate the elongation at break and tensile strength of EVA/120 phr HH (Ultracarb LH15X) formulations as a CNT or CB content function. They were not the same as for the MH Magnifin A-H10A and MH KISUMA 5B formulations; distinct tendencies were found for other CNTs despite the comparatively low content. The elongation at break reduced somewhat with increasing CNT or CB content in the CNT50, CNT75, and CB formulations throughout a range of up to a 4 phr content. In the CM-95 formulation, the elongation at break decreased with the increase in content. However, the tensile strength did not change with the increase in the CNT or CB content, and almost the same trends were observed for the four formulations. The mechanical properties of the MH Magnifin A-H10A formulations differed significantly from those of the MH KISUMA 5B formulations. The effects of the CM-95 formulation were different from those of the CNT50, CNT75, and CB formulations.

Moreover, the obtained tensile strength values were shallow at under 10 MPa in all contents of CNT or CB. From all tests of the relationships between each flame retardant and CNT/CB formulation, it was found that MH (Magnifin A-H10A) only showed the strongest mechanical properties among the various flame retardants in the CNT/CB formulations. In addition, CNT50 offered the best mechanical properties for the multiple types of CNTs. The favorable polymer–filler interaction could explain the increase in tensile strength and elongation at break. The reduction in both mechanical properties can be primarily attributed to a further rise in other CNTs or the CB network density, or the aggregation of particles and stress concentration. Furthermore, if the composite has a high interfacial area and a strong interfacial interaction between the fillers and the polymer matrix, the third phase as an interphase is generated [38,39]. This interphase dramatically influences the mechanical properties of polymer composites. We did not use functionalized fillers in the examined EVA composites, which could account for the enhanced interaction between them and the matrix molecules. A lack of interaction and poor wettability of the employed CNT/75, CM-95, or CB systems in EVA may significantly minimize polymer wrapping around CNTs and CB, influencing their tensile capabilities.

**Figure 9:**
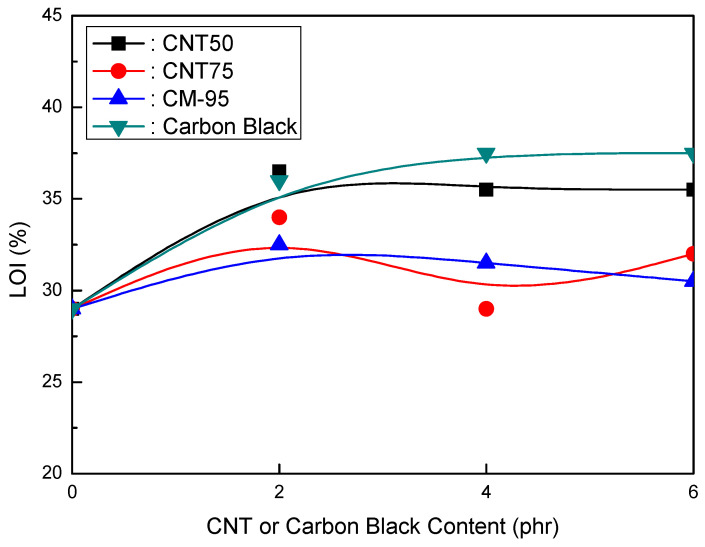
LOI (%) of EVA/120 phr Ultracarb LH15X formulations as a function of CNT or CB content.

The LOI (%) of the EVA/120 phr HH (Ultracarb LH15X) formulations as a CNT or CB content function is shown in Figure 9. Different findings were obtained for the MH Magnifin A-H10A and MH KISUMA 5B formulations; the CNT50 and CB formulations demonstrated more flame retardancy than the CNT75 and CM-95 formulations. The flame retardancy increased with increasing CNT or CB contents up to 2 phr contents and then stayed constant at higher content levels. CNT50 and CB consistently showed increased flame retardancy with combinations of various flame retardants in all tested/evaluated interactions between each flame retardant and CNT/CB composition. We discovered that a filler mix system including MH (Magnifin A-H10A) as the primary flame retardant combined with CNT50 had the optimum synergistic effect.

### 3.3. Effects of CNT50 Contents on Flame-Retardant EVA/LLDPE Composites

From the study of the relationships between each flame retardant and CNT/CB, it was found that the CNT50 formulations had the best results in terms of their mechanical properties and flame retardancy. CNT50 was the ideal composition length and size, with clean flame retardants included. CNTs with an outer diameter of 40–60 nm and a length distribution of less than 20 m can be substituted for CB in thermoplastic, clean, flame-retardant compositions to improve their mechanical and flame-retardant properties. Consequently, CNT50 was chosen for a thorough analysis in comparison to CB (Section 3.1). The effects of CNT50 on various types of flame-retardant materials were investigated.

**Figure 10:**
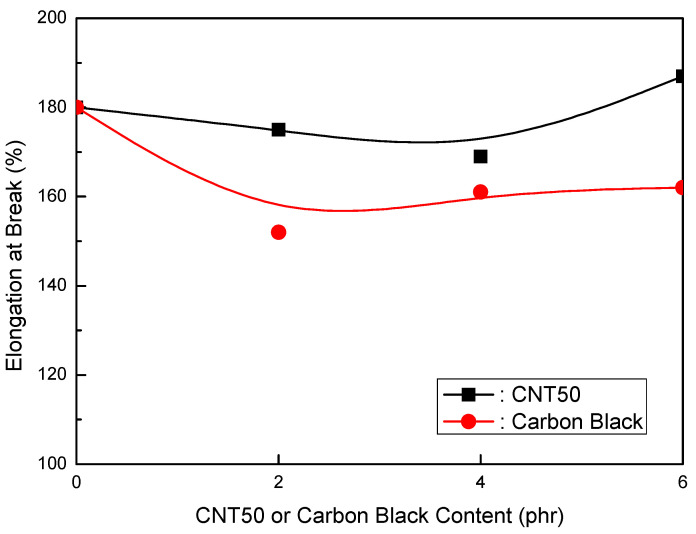
Elongation at break (%) of EVA/LLDPE/120 phr (Magnifin A-H10A) formulations as a function of CNT or CB content.

**Figure 11:**
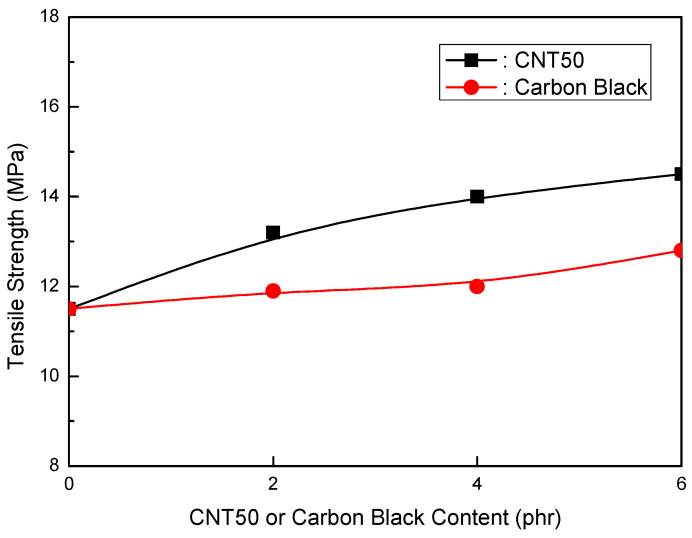
Tensile strength of EVA/LLDPE/120 phr (Magnifin A-H10A) formulations as a function of CNT or CB content.

**Figure 12:**
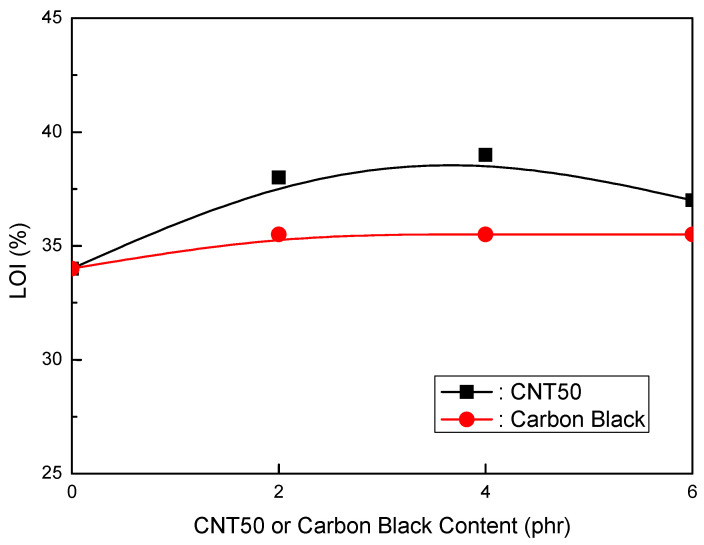
LOI (%) of EVA/LLDPE/120 phr (Magnifin A-H10A) formulations as a function of CNT or CB content.

Formulations of EVA/LLDPE/MH (Magnifin A-H10A) (120 phr)/CNT50 were tested. Table 8 shows the formulations in detail. The mechanical properties of the CNT50 formulations were higher than those of the CB formulations, as seen in Figure 10 and Figure 11. When the CNT50 content increased, the elongation at break and CB content increased slightly. Furthermore, the tensile strength increased as the content of CNT50 or CB increased, but at a faster pace than the CB content. In particular, CNT50 outperformed CB in terms of both mechanical properties. These findings indicate that it may be an acceptable flame retardant if CNT50 can attain the same or better flame retardancy than thermoplastic clean flame-retardant formulations. Surprisingly, CNT50 formulations had stronger flame retardancy than CB formulations, as illustrated in Figure 12. The flame retardancy increased as the CNT50 or CB content increased. The CNT50 formulations grew at a faster rate than the CB formulations. Furthermore, the volume resistivity of the CNT50 and CB formulations is more significant than 1 × 1015 Ωcm, making them suitable for usage in jacket materials and wire and cable insulation.

These findings are consistent with earlier research findings that the electrical properties of a polymer are proportional to its size [44]. As a result, larger sizes result in worse electrical characteristics. The electrical characteristics deteriorated as the size/volume decreased. However, when the size was substantial, the electrical characteristics were strong. Small-sized polymers have low electrical properties, specifically electrical resistivity, whereas large-sized polymers have greater volume resistivity [45]. Results show that the electric resistivity of a polymer varies according to the thickness of the layer [46]. Because of its electric volume resistivity, the thickness and size of a polymer are significant when choosing one. Volume resistivity is a type of electrical insulation. According to the overall mechanical properties, flame retardancy, and electrical properties of CNT50/CB formulations, CNT50 is a suitable material for thermoplastic, clean, flame-retardant compositions instead of CB.

The experimental results confirmed the main points of this study. CNTs with an outer diameter of 40–60 nm and a length distribution of less than 20 nm can be used instead of CB in thermoplastic, clean, flame-retardant compositions to improve mechanical properties and flame retardancy. Another significant difference between clean, flame-retardant materials and routine thermoplastics is that the extruding temperature of clean, flame-retardant materials is 160–200 °C, whereas that of routine thermoplastics is 200–250 °C. Because clean, flame-retardant materials are primarily composed of low-softening-temperature-grade polymers such as EVA, ethylene alpha-olefin, or ethylene ethyl acrylate, the extruding temperature of clean flame-retardant materials is lower than that of routine thermoplastics such as polyethylene. Sheets of test specimens for mechanical properties and flame retardancy were prepared via hot pressing and compressing at 180 °C for 10 min with a thickness of 2 mm. The above materials are preferably extruded from 160 °C to 200 °C onto conductors to prepare the insulated cable and check the processability. This extruding method is identical to the standard thermoplastic method. During the cable extrusion of the above compositions, the non-CNT50 content composition (M-1) and the 2–4 phr CNT50 composition (M-2 and M-3) demonstrated the finished cables’ best processability and excellent surface smoothness.

Table 9 shows the results of the EVA/LLDPE/MH (KISUMA 5B)/CNT50 formulations. The non-CNT50 content composition (run number M-5) and the 2–4 phr CNT50 content composition (run numbers M-6 and M-7) demonstrated the best processability and final cable surface smoothness. As indicated in Table 10, intumescent flame retardants such as RP, ZB, and BA were utilized in the EVA/LLDPE/MH (MAGNIFIN A-H10A) formulations to increase their flame retardancy and achieve V-0 of the UL94 test. The test specimen and cable extrusion operations were unchanged from previous methods.

These findings indicate that the compositions of 2–4 phr of CNT50 (run numbers M-10 and M-11) exhibit good mechanical and flame-retardant qualities. In particular, all compositions satisfy the UL94 test’s V-0 requirement, and their volume resistivity is sufficient for use as an insulating material for wire and cable. At 2–4 phr of CNT50 content, the elongation at break of run numbers M-10 and M-11 were somewhat diminished. Table 4 illustrates how the elongation at break was significantly reduced when CB was utilized instead of CNT50 in run numbers M-10 and M-11. Furthermore, every compound passed the thermal aging test (100 °C × 136 h), demonstrating exceptional thermal characteristics. For completed cables, the non-CNT50 content composition (run number M-9) and the 2–4 phr CNT50 compositions (run numbers M-10 and M-11) exhibited the best processability and superior surface smoothness.

## 4. Conclusions

A synergistic effect of CNTs or CB in formulations based on non-halogenated flame retardants and polymeric matrices of different natures was investigated to obtain HFFR compounds. The present study provides an optimal recipe for formulating polymer composites with the necessary flame-retardant and mechanical properties in cable and wire applications.

1. EVA/120 phr MH (Magnifin A-H10A)/CNT or CB Formulations

It is proposed that particle size influences mechanical characteristics. When the mechanical properties (elongation at break and tensile strength) of the CNT50 formulation and the CB formulation are examined, it is clear that the CNT50 formulation outperforms the CB formulation.

2. EVA/LLDPE/120 phr MH (Magnifin A-H10A)/CNT or CB Formulations

The CNT50 formulations showed better mechanical properties than the CB formulations. The elongation at break slightly increased with the increased CNT50 content, while the elongation at break slightly decreased with the increase in CB content. Furthermore, the tensile strength increased with an increase in CNT50 or CB. However, the increasing rate of the CNT50 content was higher than that of the CB content. For both mechanical properties, higher values were obtained for CNT50 than CB. These results are exciting, and CNT50 can be a suitable flame retardant for practical HFFR formulations if CNT50 accepts higher flame retardancy. In addition, higher flame retardancy was observed in the CNT50 formulations than in the CB formulations. According to the overall results of the mechanical properties and flame retardancy of CNT/CB formulations, CNT50 is a suitable flame retardant in HFFR formulations and improves mechanical properties and flame retardancy.

## Figures and Tables

**Table 1:** Formulations of EVA/LLDPE/120 phr flame-retardant MH (Magnifin A-H10A) as a function of CB content.

Content ^a^/Property	C-1	C-2	C-3	C-4	C-5
CB (phr)	0	2	4	6	8
Tensile strength (MPa)	9	9	9.5	9.5	10
Elongation at break (%)	165	150	150	150	145
LOI (%)	31	33	34	37	39
UL-94 test	H-B	H-B	H-B	H-B	H-B

^a^ In addition, formulations contain EVA (80%); LLDPE (20%); MH (Magnifin A-H10A) (120 phr); and Irganox1010 antioxidant (1 phr).

**Table 2:** Formulations of EVA/LLDPE/120 phr flame-retardant HH (Ultracarb LH15X) as a function of CB content.

Content ^a^/Property	C-6	C-7	C-8	C-9	C-10
CB (phr)	0	2	4	6	8
Tensile strength (MPa)	7	7	8	9	9
Elongation at break (%)	160	145	140	130	130
LOI (%)	27	32	34	35	37
UL-94 test	H-B	H-B	H-B	H-B	H-B

^a^ In addition, formulations contain EVA (80%); LLDPE (20%); HH (Ultracarb LH15X) (120 phr); and Irganox1010 antioxidant (1 phr).

**Table 3:** Formulations of EVA/LLDPE/120 phr flame-retardant MH (KISUMA 5B) as a function of CB content.

Content ^a^/Property	C-11	C-12	C-13	C-14	C-15
CB (phr)	0	2	4	6	8
Tensile strength (MPa)	10	9.5	9.5	9.5	9.5
Elongation at break (%)	550	500	481	450	420
LOI (%)	27	31	32	32	35
UL-94 test	H-B	H-B	H-B	H-B	H-B

^a^ In addition, formulations contain Evaflex 360 (80%); LLDPE (20%); MH (KISUMA 5B) (120 phr); and Irganox1010 antioxidant (1 phr).

**Table 4:** Formulations of EVA/LLDPE/130 phr flame-retardant MH (Magnifin A-H10A) and intumescent flame-retardant (RP + ZB + BA) as a function of CB content.

Content ^a^/Property	C-16	C-17	C-18	C-19	C-20
CB (phr)	0	2	4	6	8
Tensile strength (MPa)	10	9.5	10	10	10
Elongation at break (%)	150	130	115	105	100
LOI (%)	34	35	37	38	40
UL-94 test	V-0	V-0	V-0	V-0	V-0

^a^ In addition, formulations contain EVA (80%); LLDPE (20%); MH (Magnifin A-H10A) (130 phr); RP (5 phr); ZB (5 phr); BA (2 phr); and Irganox1010 antioxidant (1 phr).

**Table 5:** EVA/120 phr MH (Magnifin A-H10A) formulations as a function of CNT50, CNT75, CM-95, and CB content.

Content ^a^/Property	P-1	P-2	P-3	P-4	P-5	P-6	P-7	P-8	P-9	P-10	P-11	P-12	P-13
CNT50 (phr)	-	2	4	6	-	-	-	-	-	-	-	-	-
CNT75 (phr)	-	-	-	-	2	4	6	-	-	-	-	-	-
CM-95 (phr)	-	-	-	-	-	-	-	2	4	6	-	-	-
CB (phr)	-	-	-	-	-	-	-	-	-	-	2	4	6
Tensile strength (MPa)	10.3 ± 0.3	11.3 ± 0.1	11.5 ± 0.2	12.0 ± 0.2	10.7 ± 0.5	10.3 ± 0.2	11.8 ± 0.3	11.6 ± 0.6	12.0 ± 0.2	12.7 ± 0.2	9.3 ± 0.2	9.5 ± 0.1	10.8 ± 0.3
Elongation at break (%)	200 ± 25	200 ± 5	180 ± 19	185 ± 26	190 ± 20	166 ± 23	190 ± 19	164 ± 16	133 ± 7	135 ± 5	174 ± 14	151 ± 9	181 ± 5
LOI (%)	33.0	34.5	36.5	40.5	35.5	36.5	40.0	31.5	31.5	31.5	35.0	36.5	39.0

^a^ In addition, formulations contain Evaflex 360 (100%); magnesium hydroxide (H10A) (120 phr); and Irganox1010 antioxidant (1 phr).

**Table 6:** EVA/120 phr MH (KISUMA 5B) formulations as a function of CNT50, CNT75, CM-95, and CB content.

Content ^a^/Property	C-13	C-14	C-15	C-16	C-17	C-18	C-19	C-20	C-21	C-22	C-23	C-24	C-25
CNT50 (phr)	-	2	4	6	-	-	-	-	-	-	-	-	-
CNT75 (phr)	-	-	-	-	2	4	6	-	-	-	-	-	-
CM-95 (phr)	-	-	-	-				2	4	6	-	-	-
CB (phr)	-	-	-	-	-	-	-	-	-	-	2	4	6
Tensile strength (MPa)	10.2 ± 0.3	10.0 ± 0.3	10.0 ± 0.7	8.3 ± 0.5	10.1 ± 0.3	8.6 ± 0.4	8.9 ± 0.2	9.1 ± 0.8	7.3 ± 0.5	7.0 ± 0.4	10.4 ± 0.2	9.8 ± 0.2	9.6 ± 0.4
Elongation at break (%)	705 ± 14	702 ± 10	736 ± 29	662 ± 44	725 ± 22	709 ± 22	686 ± 11	571 ± 20	517 ± 17	496 ± 17	703 ± 12	697 ± 33	703 ± 19
LOI (%)	29.6	34.5	35.5	39.5	34.5	36.5	40.0	33.0	30.0	30.0	35.5	36.5	36.5

^a^ In addition, formulations contain Evaflex 360 (100%); magnesium hydroxide (KISUMA 5B) (120 phr); and Irganox1010 antioxidant (1 phr).

**Table 7:** EVA/120 phr HH (Ultracarb LH15X) formulations as a function of CNT50, CNT75, CM-95, and CB content.

Content ^a^/Property	C-26	C-27	C-28	C-29	C-30	C-31	C-32	C-33	C-34	C-35	C-36	C-37	C-38
CNT50 (phr)	-	2	4	6	-	-	-	-	-	-	-	-	-
CNT75 (phr)	-	-	-	-	2	4	6	-	-	-	-	-	-
CM-95 (phr)	-	-	-	-				2	4	6	-	-	-
CB (phr)	-	-	-	-	-	-	-	-	-	-	2	4	6
Tensile strength (MPa)	8.1 ± 0.1	7.9 ± 0.1	8.3 ± 0.3	8.1 ± 0.2	7.7 ± 0.1	7.5 ± 0.1	8.1 ± 0.1	7.6 ± 0.2	7.3 ± 0.1	8.3 ± 0.2	7.4 ± 1.7	9.1 ± 0.2	9.1 ± 0
Elongation at break (%)	179 ± 16	147 ± 6	133 ± 4	127 ± 20	164 ± 11	159 ± 12	135 ± 14	92 ± 26	79 ± 5	76 ± 4	162 ± 22	121 ± 17	166 ± 30
LOI (%)	29.0	36.5	35.5	35.5	34.0	29.0	32.0	32.5	31.5	30.5	36.0	37.5	37.7

^a^ In addition, formulations contain Evaflex 360 (100%); HH (Ultracarb LH15X) (120 phr); and Irganox1010 antioxidant (1 phr).

**Table 8:** EVA/LLDPE/120 phr flame-retardant MH (Magnifin A-H10A) formulations as a function of CNT50 content.

Content ^a^/Property		M-1	M-2	M-3	M-4
CNT50 (phr)		0	2	4	6
Tensile strength (MPa)		12	13	14	14
Elongation at break (%)		180	175	170	170
Thermal aging at 100 °C for 168 h	Retention of tensile strength (%)	Over 80%			
	Retention of elongation at break (%)	Over 80%			
LOI (%)		34	38	39	39
UL-94 test		H-B	H-B	H-B	H-B
Volume resistivity (Ωcm)		4 × 10^15^	2 × 10^15^	2 × 10^15^	1 × 10^15^

^a^ In addition, formulations contain EVA (80%); LLDPE (20%); MH (Magnifin A-H10A) (120 phr); and Irganox1010 antioxidant (1 phr).

**Table 9:** EVA/LLDPE/120 phr flame-retardant MH (KISUMA 5B) formulations as a function of CNT50 content.

Content ^a^/Property		M-5	M-6	M-7	M-8
CNT50 (phr)		0	2	4	6
Tensile strength (MPa)		10	10	10	10
Elongation at break (%)		550	500	450	440
Thermal aging at 100 °C for 168 h	Retention of tensile strength (%)	Over 80%			
	Retention of elongation at break (%)	Over 80%			
LOI (%)		27	30	33	35
UL-94 test		H-B	H-B	H-B	V-2
Volume resistivity (Ωcm)		3 × 10^15^	3 × 10^15^	1 × 10^15^	1 × 10^15^

^a^ In addition, formulations contain EVA (80%); LLDPE (20%); MH (KISUMA 5B) (120 phr); and Irganox1010 antioxidant (1 phr).

**Table 10:** EVA/LLDPE/130 phr flame-retardant MH (Magnifin A-H10A) and intumescent flame-retardant (RP + ZB + BA) formulations as a function of CNT50 content.

Content ^a^/Property		M-9	M-10	M-11	M-12
CNT50 (phr)		0	2	4	6
Tensile strength (MPa)		10	11	11	11.5
Elongation at break (%)		150	145	145	140
Thermal aging at 100 °C for 168 h	Retention of tensile strength (%)	Over 80%			
	Retention of elongation at break (%)	Over 80%			
LOI (%)		34	36	38	39
UL-94 test		V-0	V-0	V-0	V-0
Volume resistivity (Ωcm)		2 × 10^15^	1 × 10^15^	2 × 10^15^	9 × 10^15^

^a^ In addition, formulations contain EVA (80%); LLDPE (20%); MH (Magnifin A-H10A) (130 phr); RP (5 phr); ZB (5 phr); BA (2 phr); and Irganox1010 antioxidant (1 phr).

## Data Availability

Data are contained within the Article and Supplementary Materials.

## Supplementary Materials

The following supporting information can be downloaded at: https://www.mdpi.com/article/10.3390/polym16030417/s1, Figure S1: SEM micrographs of CNTs taken by SEM system, Model NNL200 from FEI Company, Eindhoven, Netherlands.
