# Supplementary material for: A Systematic Investigation on the Effect of Carbon Nanotubes and Carbon Black on the Mechanical and Flame Retardancy Properties of Polyolefin Blends
Source: Polymers (Basel). 2024 Feb 1;16(3):417. doi: 10.3390/polym16030417 (PMC10856896; doi:10.3390/polym16030417)
Supplement: Supplementary file 1 [file polymers-16-00417-s001.zip › polymers-2754820-supplementary.pdf]

A Systematic Investigation on the Effect of Carbon Nanotubes and Carbon Black on the Mechanical and Flame Retardancy of Polyolefin Blends

Eid M. Alosime [1,*] and Ahmed A. Basfar [2,3]

[1] King Abdulaziz City for Science and Technology, P.O. Box 6086, Riyadh 11442, Saudi Arabia
[2] M.Sc. in Nuclear Engineering Program, College of Engineering, King Saud University, Riyadh P.O. Box 145111, Saudi Arabia; abasfar@ksu.edu.sa
[3] Mechanical Engineering Department, College of Engineering, King Saud University, P.O. Box 800, Riyadh 11421, Saudi Arabia
* Correspondence: alosimi@kacst.gov.sa

In thermoplastic clean flame-retardant compositions, the compatibility of CNTs with various tube lengths and diameter sizes is examined. The following materials are being investigated to determine which is more compatible with the other components: CM-95 (Hanwha Nanotech/Korea, multi-walled CNT synthesized by catalytic CVD process, diameter range: 10-15 nm); CNT 50 (NanoKarbon, Korea nano Ind. Co., multi-walled CNT synthesized by catalytic CVD process, inner diameter: 10-30 nm, outer diameter: 40-60 nm, length distribution: under 20 μm); and CNT 75 (NanoKarbon, Korea nano Ind. Co., multi-walled carbon nanotubes synthesized by catalytic CVD process, inner diameter: 30-50 nm, outer diameter: 60-80 nm, length distribution: under 20 μm). Figure S1 displays the SEM micrographs of the CNTs used in this investigation.

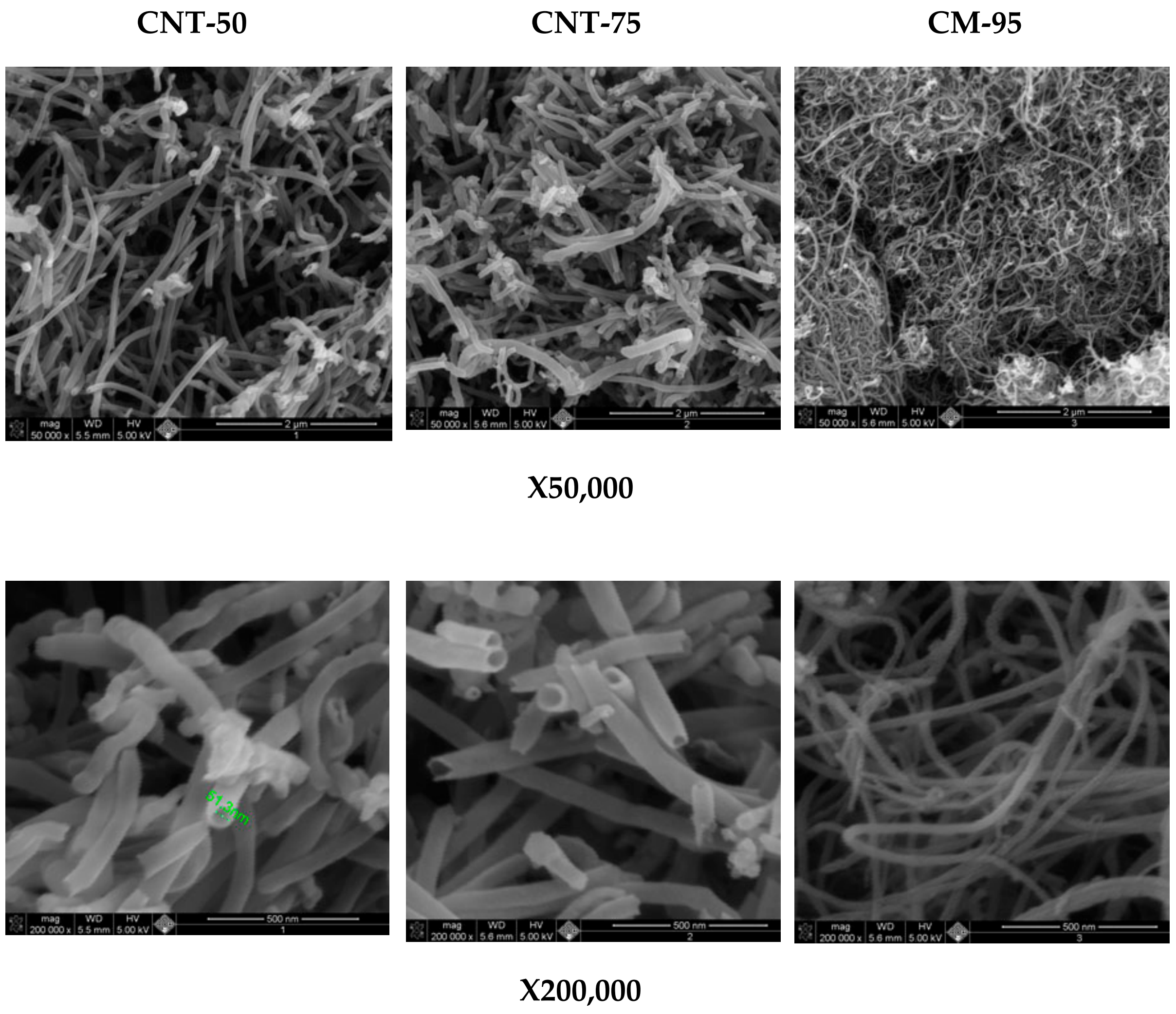

Figure S1. SEM micrographs of CNTs taken by SEM system, Model NNL200 from FEI Company, Netherlands.
